# Trihelix Transcription Factor *ZmThx20* Is Required for Kernel Development in Maize

**DOI:** 10.3390/ijms222212137

**Published:** 2021-11-09

**Authors:** Peng Li, Zhaoxia Li, Guangning Xie, Juren Zhang

**Affiliations:** Key Laboratory of Plant Development and Environment Adaptation Biology, Ministry of Education, School of Life Sciences, Shandong University, Qingdao 266237, China; lipengjn18@163.com (P.L.); zhaoxia_1019@126.com (Z.L.); goldmmn@126.com (G.X.)

**Keywords:** *Zea mays*, kernel development, trihelix transcription factor, storage proteins

## Abstract

Maize kernels are the harvested portion of the plant and are related to the yield and quality of maize. The endosperm of maize is a large storage organ that constitutes 80–90% of the dry weight of mature kernels. Maize kernels have long been the study of cereal grain development to increase yield. In this study, a natural mutation that causes abnormal kernel development, and displays a shrunken kernel phenotype, was identified and named “*shrunken 2008* (*sh2008*)”. The starch grains in *sh2008* are loose and have a less proteinaceous matrix surrounding them. The total storage protein and the major storage protein zeins are ~70% of that in the wild-type control (WT); in particular, the 19 kDa and 22 kDa α-zeins. Map-based cloning revealed that *sh2008* encodes a GT-2 trihelix transcription factor, *ZmThx20*. Using CRISPR/Cas9, two other alleles with mutated *ZmThx20* were found to have the same abnormal kernel. Shrunken kernels can be rescued by overexpressing normal *ZmThx20*. Comparative transcriptome analysis of the kernels from *sh2008* and WT showed that the GO terms of translation, ribosome, and nutrient reservoir activity were enriched in the down-regulated genes (*sh2008*/WT). In short, these changes can lead to defects in endosperm development and storage reserve filling in seeds.

## 1. Introduction

Maize (*Zea mays*) is one of the most widely produced and consumed cereals worldwide. In addition to being used as human food and animal feed, it is also used as an industrial raw material for starch, oil, and other products. With a continuously growing population and the threat of climate change, the ability to ensure continuous increases in maize yields is becoming increasingly important [1]. As a large storage organ in maize, the endosperm contributes to 80–90% of the dry weight in mature maize grains [2,3] and consists of 8–11% of the protein and 72–73% of the starch in the kernel [4]. Increasing the yield of maize and improving the quality (i.e., amino acid content, lysine and tryptophan content, and starch/amylose/amylopectin content) have always been the focus of crop science. During the 1–4 days after pollination (DAP), the endosperm comprises a large coenocyte in which nuclear divisions occur without cellularization. At approximately 6 DAP, the endosperm differentiates into starchy endosperm, the basal endosperm transfer layer, the aleurone layer, and the embryo-surrounding region [2,5,6]. In the two weeks following pollination, functional tissues are shaped from single cells, readying the kernel for filling with storage compounds. In the past decades, factors like plant hormones, sugars, peptides/receptors, and transcription factors have been identified in early maize kernel development [2]. However, in the filling and dehydration stages, the mechanisms are largely unknown. It will be interesting and meaningful to understand the endosperm development and molecular regulatory networks for plant scientists and breeders.

Breeders and scientists have paid much attention to maize grain mutants because they are highly correlated with yield and quality, especially mutants related to protein and starch content. Mutations that alter starch or protein content often lead to changes in kernel size and weight. As the most abundant storage protein, zeins represent 70% of the total protein stored in maize seeds [3,7]. Abnormal levels of zein protein synthesis, cleavage, localization, and storage lead to significant morphological and content changes in the kernels. Zeins can be divided into four different subfamilies based on their solubility and structural relationships: α (19 and 22 kDa), γ (50, 27, and 16 kDa), β (15 kDa), and δ (18 and 10 kDa), and α-zeins are highly expressed in the maize endosperm [8,9]. Interestingly, when zein protein levels are low or not assembled properly, kernels show an opaque or semi-opaque phenotype, which sometimes leads to the Unfolded Protein Response (UPR). A defective signal peptide in a 19 kDa α-zein has been found to lead to Defective endosperm* (De*)-B30 [10]. Mutant kernels have an opaque, starchy phenotype and malformed zein protein bodies, wherein UPR is active. *Maize floury 4* (*fl4*) is caused by a mutated z1A 19 kDa α-zein with a defective signal peptide cleavage site, leading to semi-opaque kernels with small, misshaped, and aggregated protein bodies, along with a dilated endoplasmic reticulum (ER) [11]. *Floury2* (*fl2*) encodes 22 kDa α-zein with a defective signal peptide [9], resulting in a soft, starchy endosperm and a high lysine content in maize. Mucronate (*Mc*) has been identified as a dominant maize opaque kernel mutation with altered zein storage protein synthesis and increased expression of the UPR marker binding immunoglobulin protein (BIP) [12], due to the abnormal 16 kDa γ-zein gene with a 38-bp deletion between nucleotides 438 and 476. *Floury1* (*FL1*) encodes a transmembrane protein that is located in the protein body, ER membrane, and participates in protein body formation by facilitating the localization of 22 kDa α-zein, which is essential for the formation of the vitreous endosperm [13]. When mutated, *floury1* has been found to have a starchy endosperm and opaque kernel phenotype. *Opaque7* encodes an acyl-activating enzyme-like protein involved in storage protein synthesis in the endosperm; mutation of *Opaque7* leads to the reduction of α-zein concentrations, starchy endosperm, and opaque kernels [14]. Opaque1 (O1) and O10 do not affect the synthesis of proteins but lead to an opaque phenotype by affecting the assembly of proteins [15,16]. The abnormal localization and accumulation of certain zein proteins in the endosperm have been observed in these mutants. Other metabolic and cellular processes have also been found to shape an opaque phenotype. *O5* encodes a monogalactoside diacylglycerol synthase (MGD1), which causes an opaque phenotype by affecting the amyloplast membrane around starch grains [17]. Moreover, *Floury 3* (*FL3*) encodes a PLATZ protein that interacts with two critical factors of the RNA polymerase III (RNAPIII) transcription complex, RNA polymerase III subunit 53 (RPC53), and transcription factor class C 1 (TFC1), participating in transcriptional regulation of tRNA and 5S rRNA and regulating endosperm development [18].

Evidence has shown that the altered transcriptional regulation of zein protein synthesis and processes will lead to similar maize kernel phenotypes, including starchy endosperm, opaque kernels, changed amino acid content, and increased UPR. This has helped to identify potential upstream transcriptional regulators in endosperm development and quality control. Some of the classical opaque mutants were identified as mutations in transcription factors, such as Opaque2 (O2). O2 is a DNA-binding protein belonging to the bZIP transcription factor that recognizes specific target sites and activates downstream target genes, including 14 kDa β-zein, 10 kD a-zein, 19 kD a-zein, and 22 kDa α-zein [19,20,21,22,23,24]. *O2* kernels contain over 70% higher lysine content than wild-type kernels [25]. The other two bZIP proteins (O2-heterodimerizing proteins 1 (OHP1) and OHP2) were identified as interactors of O2 to form the homodimerize or heterodimerize protein complex that binds to the O2-box in zein gene promoters and then activate their expression [26,27,28,29]. O2 has been shown to physically interact with the DOF family TF Prolamin-box binding factor (PBF), recognize a motif containing the AAAG (P box) and ACGT (O2 box) core elements, and regulate the expression of pyruvate orthophosphate dikinase 1 and 2 (PPDK1and PPDK2), and starch synthase III (SSIII) genes [30]. The MADS-box protein, ZmMADS47, has been identified as an O2 interacting protein, and when both O2 and ZmMADS47 are present, the transactivation of the promoters (α-zein classes z1A, z1B, z1C, z1D, and 50 kDa γ-zein genes) was greatly enhanced because O2 released the transactivation ability of ZmMADS47 [31]. O2 has also been shown to interact with the transcriptional adaptor alteration/deficiency in activation 2 (ADA2) protein and the histone acetyltransferase GCN5 to regulate O2 target gene expression. An O2 negative regulator, Taxilin protein, has been identified as a regulator of zein gene expression [32]. Taxilin could change the subcellular distribution of O2 and significantly change the repressed transcriptional activity of O2 on the 22 kDa zein promoter. In addition to the O2-dependent transcriptional regulation of opaque-related genes, transcription factors ZmbZIP22 [33] and ZmNAC128/130 [34] have been identified to contribute to the transcriptional regulation of genes coding for storage protein and/or starch synthesis genes. ZmNAC128 and ZmNAC130 function as transcriptional activators of the 16 kDa γ-zein genes and Bt2 (brittle2, adenosine diphosphate glucose pyrophosphorylase (AGPase) small subunit) [34].

In addition to opaque endosperm mutants, which commonly exhibit opaque, floury kernels, another well-known maize mutant is the “shrunk” kernels. Interestingly, changes in sucrose and starch synthesis, transport, and accumulation were the major reasons for the shrunk kernels. Six loci corresponding to kernel starch synthesis genes (*shrunken1* (*sh1*), *sh2*, *brittle endosperm2*(*bt2*), *amylose extender1* (*ae1*), *sugary1*, and *waxy1*) and are well-known for starch and yield-related traits. The *shrunken1* (*sh1*) mutants have a shrunken kernel phenotype because sucrose synthetase, which converts UDP-glucose to sucrose, is inactivated [35]. The *shrunken 2* (*sh2*) and *bt2* mutants encode the ADP-glucose pyrophosphorylase large and small subunits, respectively, which coordinate the conversion of UDP-glucose to ADP-glucose [2]. ADP-glucose is then converted to amylose by granule-bound starch synthetase encoded at *Wx1* [36,37] or to amylopectin by starch branching enzyme IIB and debranching enzyme encoded at *ae1* and *su1* [38,39]. Sweet corn is the kernel of *sugary1* (*su1*) mutant [40], which accumulates sucrose with a shrunken or wrinkled appearance of dry kernels, whereas *wx1* mutants do not synthesize amylose; thus, a waxy genotype is amylose-free [36,41]. The *miniature1* (*mn1*) seed phenotype in maize is caused by a loss-of-function mutation at the *mn1* locus, which encodes a cell wall invertase (INCW2) that localizes exclusively to the basal endosperm transfer cells (BETCs) of developing seeds [42].

Trihelix transcription factors are plant-specific transcription factors that were initially called GT factors because they bind to light-responsive GT elements [43]. The DNA-binding domain of the GT factor, rich in basic and acidic amino acids and proline and glutamine residues, features a typical trihelix (helix-loop-helix-loop-helix) structure that determines the specific binding of GT elements [44]. The interaction between GT and GT elements has been implicated in the complex transcriptional regulation of many plant genes. The SANT/myb domain of Trihelix transcription factors is a motif of ~50 amino acids involved in chromatin remodeling and transcription regulation. In Arabidopsis, the GT1 subfamily genes participate in salt stress and pathogen responses [45]. Trihelix transcription factors are also associated with plant morphogenesis [44]. There are 57 members of the Trihelix family in maize (http://planttfdb.gao-lab.org/family.php?sp=Zma&fam=Trihelix (accessed date 8 November 2021)). No detailed functional analysis of the maize Trihelix family has been conducted as an important transcription regulator in plant abiotic stress response and development.

In this study, a natural variation mutant that causes abnormal kernel development, and displays a shrunken kernel phenotype, was identified and named “*shrunken 2008* (*sh2008*)”. In these shrunken kernels, starch showed a significant reduction, and the total storage protein and major storage protein zeins were ~70% that of the wild-type control. The starch grains in *sh2008* were loose and had a less proteinaceous matrix surrounding them. Positional cloning and validation by gene manipulation revealed that *sh2008* is caused by a mutation of GRMZM2G169580, a GT-2 trihelix transcription factor. The shrunken kernel was rescued by the overexpression of normal *ZmThx20*. These results highlight the potential role of ZmThx20 in mediating transcriptional regulation and mechanism, particularly in storage proteins, starch accumulation, and endosperm development.

## 2. Results

### 2.1. Identification of Maize sh2008 Mutant with Distorted and Shrunken Kernels

**Figure 1:**
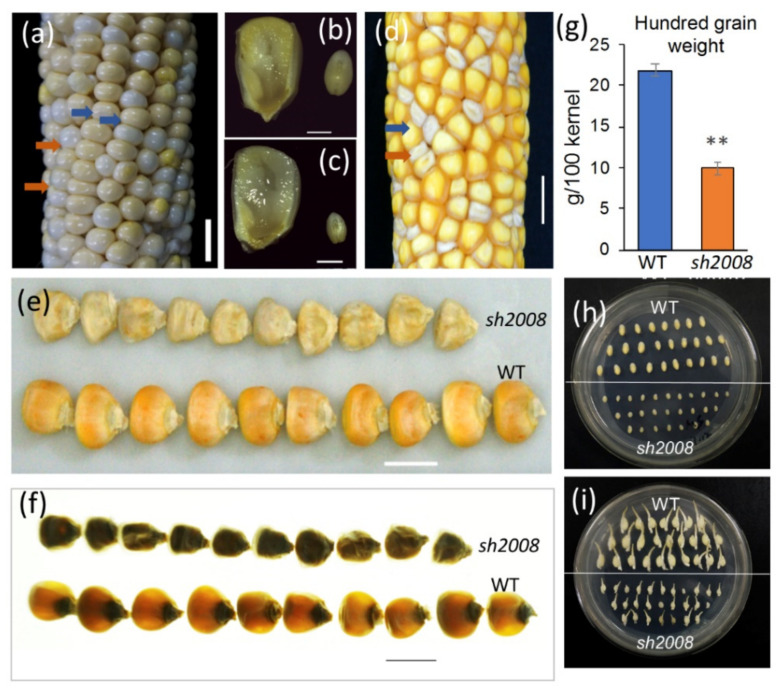
Kernel phenotype of the *sh2008* mutant and WT. (**a**) The F2 ears at 14 DAP. Blue arrows indicate the normal kernels, which the content from fluid to milky white fluid, while the mutants (orange arrows) are still blisters and contain clear fluid. (**b**,**c**) Longitudinal section of immature WT (**b**) and *sh2008* (**c**) kernels at 14 DAP to show the content in the kernels of the mutant and WT. (**d**) The morphology of the mature heterozygous ears. Blue and orange arrows in panel (**d**) and bars in panel (**e**) indicate the WT and the *sh2008* mutant kernels. (**e**,**f**) The kernels from a heterozygous ear under natural light (**e**) and a lightbox (**f**) showing the opaque phenotype of the kernels from the *sh2008* mutant (upper) and normal control (WT, lower). (**g**) Kernel weight of the *sh2008* and WT. Values are the means of the three replicates ± SD. ** denotes statistical significance with *p* < 0.01 by using a *t*-test. (**h**,**i**) Germination assay of the immature embryos from the WT and *sh2008* mutant on 1/2 MS medium for 0 (**h**) and 6 days (**i**) showing that embryo germination was not affected in the mutant, but the development of mutant embryos was later than the WT. Scale bar, 1 cm.

A natural variation mutant that causes abnormal kernel development, and displays a shrunken kernel phenotype, was identified in the maize inbred line DH4866 (Shandong Denghai Seeds Co., Ltd., Yantai, China) and named shrunken kernel (*sh2008*). The differences between the wild-type (WT) and mutant kernels could be distinguished from the transmission of the kernel blister stage (approximately 10 to 14 DAP (days after pollination)) to the kernel milk stage (18 to 22 DAP). As shown in Figure 1a, at 14 DAP, the WT kernels began to turn light yellow, and the content changed from clear fluid to a milky white fluid, while the mutant kernels were still whitish blisters on the cob (Figure 1a). Embryo development in *sh2008* was delayed compared to that in the WT (Figure 1b,c). The starch in the endosperm was not fully filled in the pericarp of *sh2008*, and a cavity in the middle of the kernel was observed. When the milky inner fluid changed to a doughy consistency, the phenotype of collapse at the top of the mutant kernel appeared (Figure 1d). When harvested, the mutant kernels were smaller than the WT and had an obvious opaque phenotype under a lightbox (Figure 1e,f). The 100-kernel weight of mutant seeds was only 10 g, accounting for 47% of the WT grain (Figure 1g). To test the viability of mutant seed embryos, immature embryos (22 DAP) collected from the WT and *sh2008* mutants were cultured on 1/2 MS medium (Figure 1h). We found that there was no difference in germination rates after six days, but the development of mutant embryos lagged behind that of the WT embryos (Figure 1i). No obvious differences were observed in the seedlings, mature plants, and pollen activity from the seeds of the *sh2008* and WT plants, except for a slightly slower growth (Figure S1).

**Figure 2:**
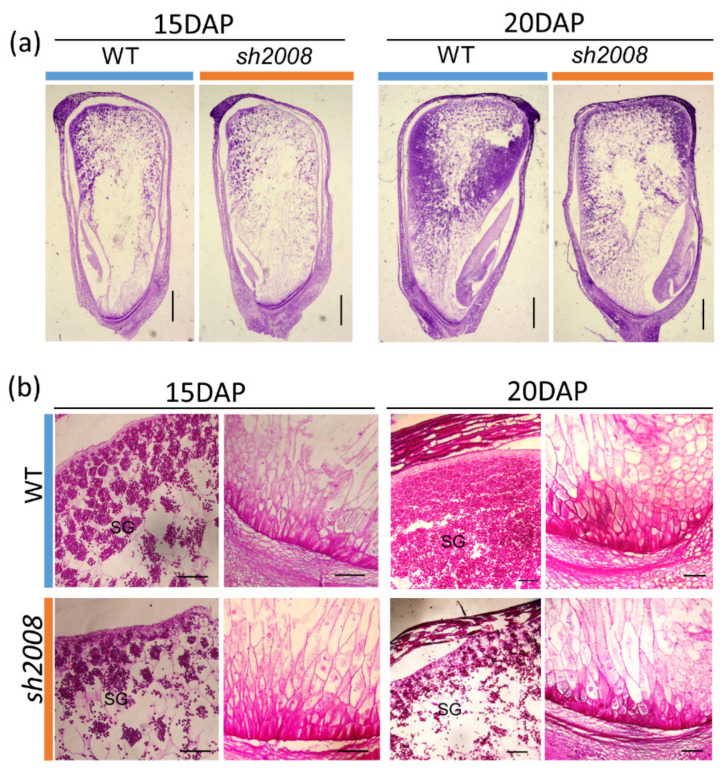
Light microscopy analysis of the immature kernel from the *sh2008* and WT at 15 DAP and 20 DAP. (**a**) Light microscopy of paraffin sections of the WT and *sh2008* kernels harvested from the same ears at 15 DAP and 20 DAP, respectively. (**b**) Microstructure of developing endosperms to show the starch granules (SG) and basal endosperm transfer cells (BETCs) of the WT and *sh2008* at 15 DAP and 20 DAP, respectively. SG, starch granule. Scale bar, 1 mm.

To further examine the differences in maize kernel development, kernels at 15 and 20 DAP were used for paraffin sections. Although the embryo development of the mutant was delayed compared to that of the WT (Figure 2a), the embryonic axis, scutellum, and several leaf primordia were well differentiated, which ensured seed germination (Figure 1h,i). Compared with WT endosperm cells, the mutant endosperm cells developed slowly, which had less starch granule accumulation, while the WT endosperm cells filled the whole grain (Figure 2b). Further observation of the transfer cells in the basal endosperm transfer layer (BETL) revealed that the area of transfer cells in the WT was larger than that of the mutant type (Figure 2b and Figure S2). The less-developed BETL cells in the *sh2008* mutant may affect the transport of nutrients from the plant to the grain, leading to endosperm retardation.

### 2.2. The sh2008 Mutant Largely Reduced the Accumulation of the Starch and Zein Protein in the Endosperm

**Figure 3:**
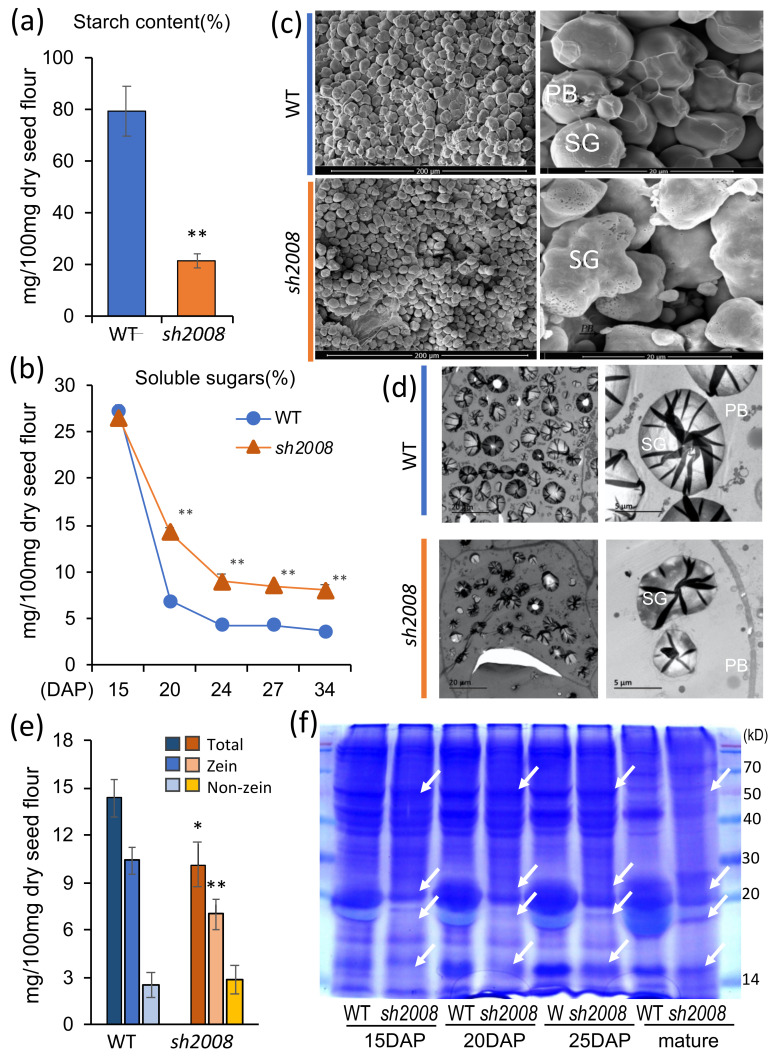
Storage carbohydrates and proteins in the WT and *sh2008*. (**a**) The starch content of the kernels from the WT and *sh2008*. The mature kernels were used for starch content measurements. Values are the means of the three replicates ± SD. ** denotes statistical significance with *p* < 0.01 by using a *t*-test. (**b**) The soluble sugars change in the WT and *sh2008* after pollination. Kernels at 15, 20, 24, 27, and 34 DAP were used for soluble sugar content measurements. Values are the means of the three replicates ± SD. ** denotes statistical significance with *p* < 0.01 by using a *t*-test when compared with the value in the WT. (**c**) Scanning electron microscopy (SEM) analysis of the peripheral regions of the mature WT and *sh2008* endosperm. SG, starch granule; PB, protein body. (**d**) Transmission electron microscopy (TEM) of the 20 DAP endosperm of the WT and *sh2008*. SG, starch granule; PB, protein body. (**e**) The content of total protein, zein protein, non-zein protein in the WT and *sh2008*. Values are the means of the three replicates ± SD. ** denotes statistical significance with *p* < 0.01 and * denotes statistical significance with *p* < 0.05 by using a *t*-test when compared with the value in the WT. (**f**) SDS-PAGE analysis of total protein, zein protein, and non-zein protein in the developing endosperms of the WT and *sh2008*. The protein mark size of each band is indicated by number. kD, kDa; DAP, days after pollination. White arrows indicate the difference between *sh2008* and WT.

To elucidate the potential reasons for the shrunken kernel phenotype of the *sh2008* mutant, two of the major components (starch and protein) in the endosperm were analyzed. The total starch content of the mutant kernel was 26% of that of the WT (Figure 3a). Possible reasons for the lower starch content in the *sh2008* mutant were a reduction of starch synthesis and/or accumulation. As the substrate of starch synthesis, the soluble sugar contents at different developmental times (15, 20, 24, 27, and 34 DAP) in the kernels from the *sh2008* mutant and WT were compared. We found that the soluble sugar contents in the *sh2008* mutant and WT were comparable at 15 DAP (approximately the end of kernel blister stage, some starch was accumulated). However, in the kernel milk stage (18–22 DAP) and kernel dough stage (24–28 DAP), the soluble sugar content was significantly higher in the *sh2008* mutant than in the WT (Figure 3b). The kernel milk stage and kernel dough stage were the two major stages of starch accumulation and contributed to the kernel weight. This could explain why the white juice flowed out of the cavity above the top when we cut the immature mutant kernels vertically, and there were fewer starch granules in the kernels at the grain filling stage.

The morphology and ultrastructure of the starch granules and protein bodies in the kernel of the *sh2008* mutant and WT were analyzed using scanning electron microscopy (SEM) and transmission electron microscopy (TEM). In the endosperm, we found that the WT starch granules were smooth and complete, arranged neatly, and surrounded by a proteinaceous matrix (Figure 3c, upper panel). In the mutants, the starch granules were loose, and a less proteinaceous matrix surrounded them. Moreover, there were many holes of different sizes on the surface of the starch granules (Figure 3c, lower panel). To observe the distribution and morphological development of protein bodies and starch granules, 20 DAP kernels were observed using TEM (Figure 3d). In WT endosperm cells, the starch granules were uniform in size, and the protein bodies were oval and distributed uniformly around the starch granules (Figure 3d, upper panel). In mutant endosperm cells, most starch granules were incomplete and variable. The protein bodies were small and unevenly distributed (Figure 3d, lower panel). Taken together, the reduced starch accumulation at the milk and kernel dough stages, and the morphology and development of starch granules and protein bodies, were altered in the kennels of the *sh2008* mutant.

Previous studies have shown that the maize opaque endosperm phenotype is often due to changes in storage protein synthesis, deposition, and metabolism, especially the zein protein [46]. To explore the cause of the opaque phenotype of our mutant kernels, we measured the changes in protein content in the mutant endosperm. Quantitative analysis showed that the total protein content decreased by 32%, and the zeins decreased by 32% compared with the WT, while the non-zeins showed no change (Figure 3e). The results of SDS-PAGE showed that the zein proteins were decreased significantly in the *sh2008* mutant endosperm, especially the 22 kDa α-zein and 19 kDa α-zein proteins (Figure 3f). We measured the free amino acid content in the mature kernels of mutant and WT plants. As shown in Figure S3, the *sh2008* mutant contains elevated levels of aspartic acid, serine, alanine, beta-alanine, and GABA. Tyrosine levels were similar in the mutant and WT. Lysine was lower in the mutant, although this difference does not seem to be significant. In maize endosperms, zein proteins reside in protein bodies (PBs) inside the ER. Changes in the accumulation of zein protein can lead to changes in the production of protein bodies and the irregular structure of protein bodies [10,11]. As shown in the TEM results, the number and shape of the protein bodies in the *sh2008* mutant were small and less frequent; this may be due to abolished zein protein accumulation changing the normal metabolism.

### 2.3. Map-Based Cloning Showed That sh2008 Encodes a Mutated ZmThx20

**Figure 4:**
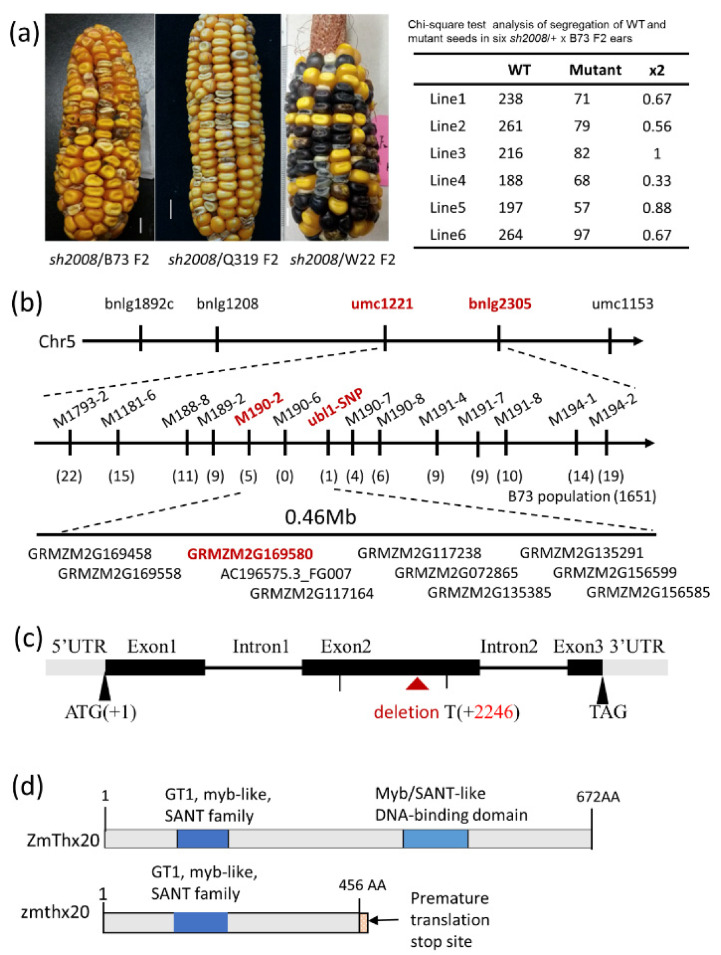
Map-based cloning showed that the *sh2008* encodes a *ZmThx20* transcription factor. (**a**) Validation of the mutant phenotype in different maize inbred lines. The *sh2008/+* were crossed with the maize lines B73, Q319, and W22; as expected, the kernels showed the same phenotype as that in DH4866. (**b**) Map-based cloning showed that the *sh2008* was roughly mapped on maize chromosome 5 L between umc1221 and bnlg2305 by Bulked-segregant analysis (BSA). Then fine mapped between markers M190-2 and 190-6 by using a population of 1651 mutant kernels from F2 ears, and candidates including *ZmThx20* (GRMZM2G169580) in this region are shown. (**c**) The gene structure of *ZmThx20* in WT and the *sh2008* is due to a 1 bp deletion in exon 2 of *ZmThx20*, which led to a premature stop codon and terminated the translation. (**d**) Protein structure and conserved domains affected by the 1 bp deletion in the *sh2008*. ZmThx20 encodes a GT2-like trihelix transcription factor. This family of transcription factors carried two DNA-binding domains (SANT/myb domain, blue indicates); however, in the *zmthx20*, the deletion of T-base (+2246 bp, based on the DNA sequence of inbred line DH4866, slightly different to B73) led to a premature stop codon and terminated translation.

The *sh2008*/+ maize line was crossed with the inbred lines B73, Q319, and W22 to produce the segregation populations with different genetic backgrounds (Figure 4a). Chi-square test of the kernels (normal:shrunken = 3:1, χ^2^ _0.05_ = 3.84, Figure 4a, right panel) from the segregation populations showed that the *sh2008* allele is a monogenic recessive nuclear mutation. Genomic DNA was extracted from 30 individuals of WT and *sh2008* plants and then mixed for the bulked pool [47]. We initially mapped the locus on the maize chromosome arm 5 L between the marker umc1221 and bnlg2305 by bulked-segregant analysis (BSA). A population of 1651 mutant kernels from F2 ears (B73 genetic backgrounds) was used for fine mapping using 23 new molecular markers (Table S1) and eventually localized *sh2008* between markers M190-2 and M190-6 (Figure 4b). Ten protein-coding genes of this section were annotated, and four genes had higher expression levels during the kernel development stage: GRMZM2G169580 (Zm00001d017420), GRMZM2G117238 (Zm00001d017423), GRMZM2G072865 (Zm00001d017424), and GRMZM2G135291 (Zm00001d017427) (Figure S4, based on [48,49]). The four gene fragments of WT and *sh2008* were amplified by PCR and sequenced. Sequencing results showed that there were several indels in the second exon of the *ZmThx20* (GRMZM2G169580, annotated as *thx20* in maize B73 RefGen_V3, V4 and NAM-5.0, named as GT-2G by Du et al., 2016 [50] and *Trihelix25* by Jiang et al., 2020 [51]. Therefore, we still use *ZmThx20* to be consistent, the prefix “*Zm*” was used to indicate the species “Zea mays” by convention), and the deletion of T-base (+2246 bp) leading to a premature stop codon and termination of translation (Figure 4c,d and Figure S5). There were no changes in the other candidate genes in this region. Therefore, GRMZM2G169580 appears to be a candidate for the *sh2008* locus.

### 2.4. Validation of the Role of ZmThx20 in Endosperm Development by Complementation Analysis and Gene-Editing

**Figure 5:**
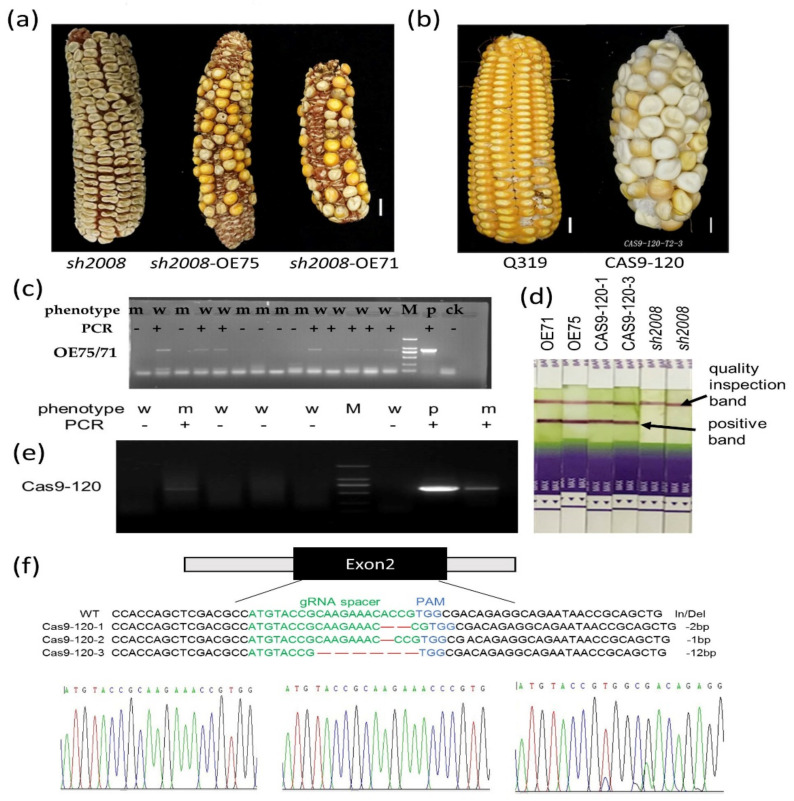
Complementation and genome-editing analysis to verify the role of *ZmThx20* in maize endosperm development. (**a**) Complementation analysis by overexpression of *ZmThx20* in the *sh2008* background. When *ZmThx20* was introduced into the *sh2008* background, the harvest T1 ears had some normal kernels as that in WT. The ORF of the *ZmThx20* was inserted into the modified plasmid vector pCAMBIA3300 (P35S::*ZmThx20*-Tnos-P35S::*bar*, P35S, CaMV35S promoter; Tnos, nos terminator; *bar*, herbicide bialaphos) and then transformed the *sh2008* mutant callus by gene gun bombardment. After herbicide screening and PCR analysis, T1 seeds were obtained from T0 plants by self-crossing. Ears from T1 seedlings were used for phenotype analysis. (**b**) Validation of the role of *ZmThx20* in endosperm development by genome-editing of *ZmThx20,* which can mimic the *sh2008* phenotype in natural mutations. (**c**,**e**) PCR analysis of the *ZmThx20* overexpression transgenic maize plants (**c**) and genome-editing plants (**e**). Lane w, wild-type control, m, PCR-positive plants. (**d**) Bar test strip analysis of the *ZmThx20* overexpression transgenic and genome-editing plants. (**f**) Sequence of the *ZmThx20* gene in transgene lines used for morphological analysis.

To determine whether *ZmThx20* is the gene responsible for the *sh2008* mutant, we integrated the open reading frame (ORF) of *ZmThx20* into the modified plasmid vector pCAMBIA3300 (P35S::*ZmThx20*-Tnos-P35S::*bar*-Tnos), and then the *sh2008* mutant callus was transformed by the gene gun bombardment method. After herbicide screening, T1 seeds were obtained from T0 plants by self-crossing (Figure 5a). The kernels that were restored to the WT phenotype were picked out and grown in soil to produce the T1 ears by self-crossing. Among them, the kernels of lines L71 and L75 showed segregating phenotypes, which were identified as transgenic events by PCR and bar test strips (Figure 5c,e). We also used the CRISPR/Cas9 system to edit the wild-type callus cells (inbred line Q319) and obtained genetically modified materials (Figure 5b). Through PCR identification and sequencing verification, the genetically modified materials were effectively edited (Figure 5d–f). As expected, the successful gene editing lines showed the same kernel phenotype as that observed in the *sh2008* mutant. Through complementation analysis and CRISPR/Cas9 editing events, we confirmed that *ZmThx20* is the gene that regulates the phenotype of grains.

### 2.5. ZmThx20 Encodes a GT-2 Like Transcription Factor and Plays Roles in Gene Expression Regulation

We constructed a phylogenetic tree based on the full-length *ZmThx20* protein and its homologs by using the Maximum Likelihood method and JTT matrix-based model (Figure S6a). The results indicated that *ZmThx20* and the other six maize trihelix transcription factors composed a *Poaceae*-specific sub-group trihelix transcription factor with the homologs in rice and sorghum. This sub-group trihelix transcription factor shared high similarity with the AtGT-2 sub-group (AtGT-2, AtDF1, and PTL) and AtGT-1 sub-group (AtGTL1, AtGT2L, and AT5G47600). *ZmThx20* has three conserved individual amphipathic α-helices and the fourth amphipathic α-helix, with the general sequence (F/Y)-(F/Y)-X-X-(L/I/M)-X-X-(L/I/M), with the exception of one less conserved tryptophan (W) in the N-terminus trihelical DNA binding domain (Figure S5), similar to other GT-2 clade members [44,52], although not the orthologs of AtGT-2 and AtGTL1. *ZmThx20* was predicted to be a member of the GT-2 trihelix family transcription factor, defined based on the features of the trihelical DNA-binding domain. The expression pattern of *ZmThx20* in various maize tissues and seed development were analyzed by qRT-PCR. As shown in Figure S6b, the expression of *ZmThx20* was detected in different organs and kernels at 8 DAP, and its expression in kernels continued later than 30 DAP, with the maximum level at 16 DAP. *ZmThx20* expression during kernel development is more modest than in roots, stems, or leaves.

### 2.6. Effects of ZmThx20 on Gene Expression

**Figure 6:**
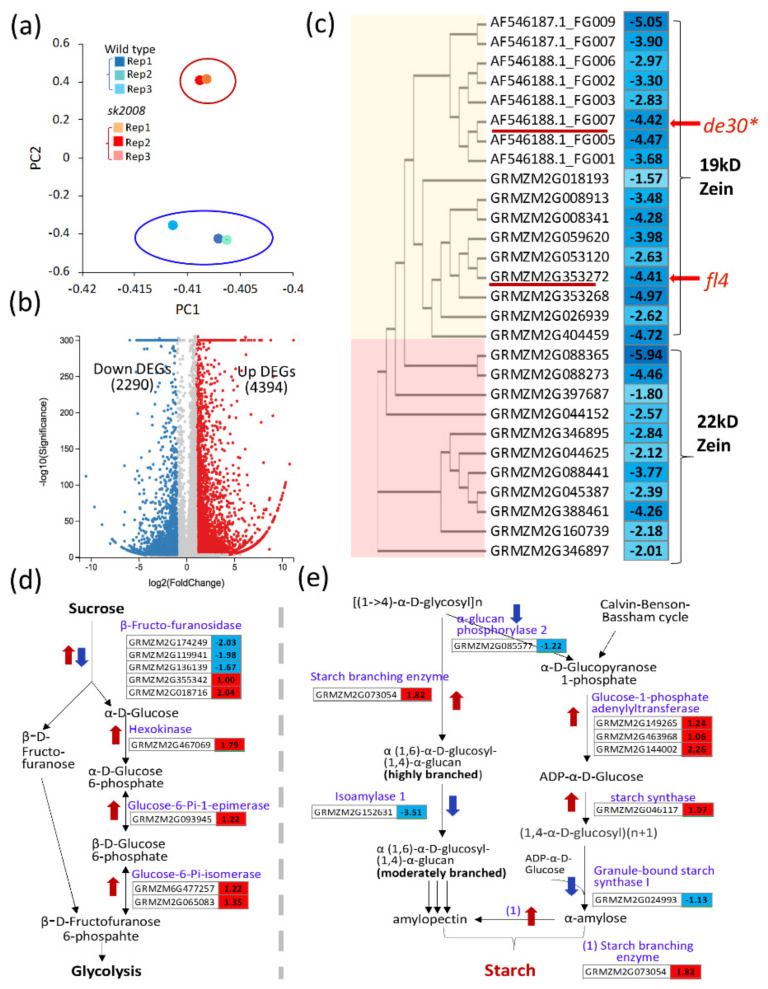
Gene expression changes in the *sh2008* compared with WT. (**a**) Principal component analysis (PCA) of data from the RNAseq analysis for the 15 DAP kernels collected from the *sh2008* mutant and WT lines can be distinguished into two groups. (**b**) Volcano plots indicate the number of DEGs that were downregulated or upregulated in comparing *sh2008* mutant to WT. DEGs were identified with Q < 0.05 and absolute log2-fold value (*sh2008*/WT) >1. (**c**) The downregulated DEGs encoding 19 kDa and 22 kDa zein proteins in comparing *sh2008* mutant to WT (*sh2008*/WT). Zein protein-coding genes were grouped into two groups based on the amino acid alignment result by using ClustalW2 (https://www.ebi.ac.uk/Tools/msa/clustalo/, accessed on 8 November 2021). The predicted translated protein sequences were based on the maize B73 genome sequence and annotation from maizeGDB (https://www.maizegdb.org, accessed on 8 November 2021) or/and EnsemblPlants (http://plants.ensembl.org/index.html, accessed on 8 November 2021). (**d**,**e**) DEGs in sucrose degradation (**d**) and starch synthesis (**e**) comparing *sh2008* and WT. Schematic of the pathways modified according to the KEGG (https://www.kegg.jp/kegg/pathway.html, accessed on 8 November 2021) and metabolic pathways at MaizeGDB (https://www.maizegdb.org/metabolic_pathways (accessed on 8 November 2021)). The color of the box represents up (red) and down (green)-regulated genes (*sh2008*/WT), and the value in the box is the log2 (*sh2008*/WT) of the genes in comparing the *sh2008* mutant to WT.

To evaluate the effects of *ZmThx20* on seed development, RNA was extracted from the 15 DAP kernels of the *sh2008* and WT. Three biological replicates were taken from each sample, and the extracted RNA was analyzed by RNAseq analysis (Figure 6a). As shown in Figure 6b and Tables S2–S6, in the comparison of the *sh2008* to WT, a total of 6684 differentially expressed genes (DEGs) consisting of 4394 upregulated and 2290 downregulated genes were identified using the Q value ≤ 0.05, and absolute Log2 (*sh2008*/WT) ≥1. The top 35 highly enriched GO terms can be divided into three different categories: response to the stimulus (consisting of 15 enriched GO terms), development (consisting of 10 enriched GO terms), and other cellular processes (consisting of 10 enriched GO terms), such as metabolic processes (Figure S7). This suggests that *ZmThx20* (or its transcriptional regulation complex) plays a role in plant development. The *ZmThx20* mutant abolished the regulation of downstream genes, including, but not limited to, the genes involved in endosperm development.

As a major storage protein of maize, zein proteins are encoded by a large multigene family whose expression pattern is tissue-specific, developmentally, and spatially regulated. In the comparison of *sh2008* and WT, 17 genes encoding 19 kDa zeins and 11 genes encoding 22 kDa zeins were downregulated in the *sh2008* mutant. In maize, the 22 kDa and 19 kDa zeins contributed to ~70% of the total zein fraction. Two of the 19 kDa zein genes Defective endosperm B30* (de30*, AF546188.1_FG007) and *floury4* (*fl4*, GRMZM2G353272) were 0.046-fold (log2 value ‒4.41) and 0.047-fold (log2 value ‒4.42) of that in WT, respectively. De*-B30 has an opaque and high lysine endosperm [10], and *fl4* is a semi-dominant opaque mutant with small, misshaped, and aggregated protein bodies [11]. The opaque endosperm and the reduced 19 kDa and 22 kDa zein proteins in *sh2008* were most likely due to the markedly downregulated zein genes. In contrast to the zeins, the metabolism of the amino acids was altered in the *sh2008*, including the alanine, aspartate, glutamate, cysteine, methionine, phenylalanine, tyrosine, and tryptophan metabolism (Table S5). For sucrose metabolism, the increased expression of enzyme-coding genes was involved in sucrose degradation (Figure 6d). For the starch synthesis pathways, the genes coding for α-glucan phosphorylase 2, granule-bound starch synthase I, and isoamylase 1 were downregulated, and isoamylase 1 was 0.088-fold (log2 value‒3.51) of that in WT. In contrast, the glucose-1-Pi adenylyltransferase, starch synthase, and starch branching enzyme coding genes were upregulated in *sh2008* (Figure 6e). Some of the zein protein-coding genes and starch synthesis genes were analyzed in the endosperm of the *sh2008* and WT by using qRT-PCR, as we saw in the transcriptome analysis, which showed that these genes were significantly changed in *sh2008* compared with WT (Figure S8).

To determine the role of *ZmThx20* in endosperm development, the expression levels of known transcription factors involved in endosperm seed development were compared with those of our *sh2008* mutant and WT (Figure S9a). Eight key factors, including O2, two PBFs, three OHPs (see details in the Introduction), ZmbZIP22, and ZmMADS47, showed different expression patterns between the *sh2008* mutant and WT. In general, they can be divided into two groups, higher in the *sh2008* mutant and lower in the *sh2008* mutant than that of the WT. The expression of O2 was significantly reduced in the *sh2008* mutant, with expression levels from 342 to 202 (FPKM, fragments per kilobase of transcript per million mapped reads) (Figure S9a). For the others, slightly reduced *PBF* (GRMZM2G146283) and *ZmMADS47* (GRMZM2G099577), and increased *PBF* (GRMZM2G146267), *OPH1* (GRMZM2G016150), *OHP1-like* (GRMZM2G019446), *OHP2* (GRMZM2G007063), and *ZmbZIP22* (GRMZM2G043063). We compared the DEGs from *sh2008* mutant versus WT and the results from *O2* [53] versus WT. As shown in Figure S9b, the *sh2008* mutant had more upregulated genes (65.74%), while more downregulated genes existed in the *o2* mutant (54.44%). Two hundred and twelve DEGs were downregulated in both the *o2* and *sh2008* mutants. They contributed to 17.2% and 22.2% of the total down- and upregulated DEGs, respectively, when compared with the WT. In *sh2008*, enrichment of the GO term nutrient reservoir activity was also observed, and 57 DEGs were involved in this GO term. In addition, 23 DEGs overlapped in both *o2* mutant and *sh2008* mutant seed storage proteins, including eight 19 kDa zein proteins, eleven 22 kDa zein proteins, one 50 kDa gamma zein, two 12S seed storage proteins (RmlC-like cupins), and one basic secretory protein. For the *O2* mutant, one GO term with significantly enriched molecular function was the nutrient reservoir activity (GO: 0045735), and 30 of the DEGs were predicted to be involved in the nutrient reservoir [20,21]. When compared with the *O2* direct target genes [20], in the *sh2008* mutant 13 of the 38 *O2* target genes were differentially expressed. However, different from the *O2* mutant, in *sh2008*, the expression changes of 19 kDa zein protein genes (both number and fold changes) were affected more significantly compared with 22 kDa zein protein genes. Together with the DEG expression changes of *O2* and *sh2008* mutants, *ZmThx20* may function through the *O2* pathway to control the expression of the zein protein genes.

### 2.7. ZmThx20 Is a Nuclear-Localized Protein and an Activator of 19 kDa Zein Gene Expression

**Figure 7:**
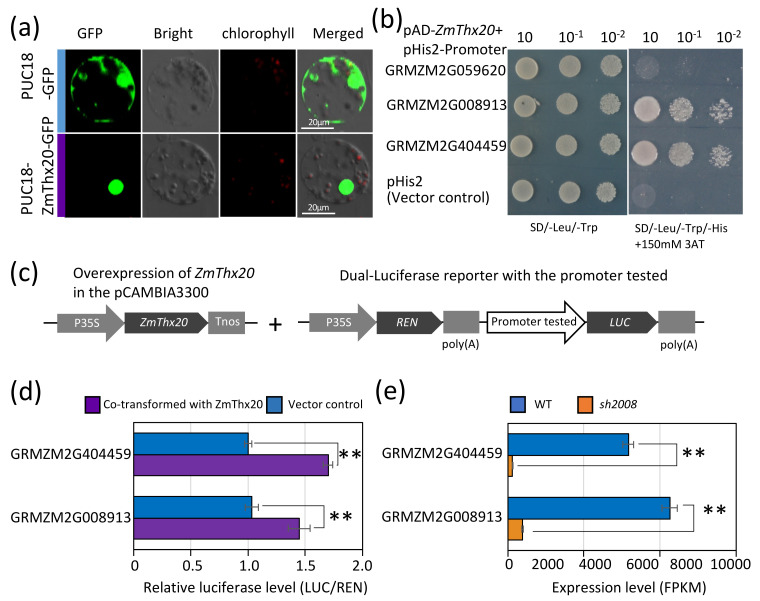
Sub-cellular localization and binding activity analysis of *ZmThx20*. (**a**) Cell localization analysis of the ZmThx20. PUC18-P35S::*ZmThx20*-GFP was used for the cell localization analysis in the transient expression system. (**b**) The growth situation of yeast YM4271 transformed with pGADT7-ZmThx20 and pHis2-19kd zein promoter on SD/-Leu/-Trp and SD/-Leu/-Trp/-His+150 mM 3AT medium. The pHis2 plasmid was used as the negative control. (**c**) Diagram of the constructs of the effector and reporter in dual-LUC reporter gene. The 35S promoter (P35S), REN luciferase (REN), firefly luciferase (LUC), and terminator (Tnos) are indicated. (**d**) Transient transcription by dual-LUC assay. The full-length ORF of *ZmThx20* was fused into the overexpression vector to produce P35S::*ZmThx20*-Tnos-P35S::*bar*-Tnos. For the reporters, the promoters of 19 kDa zein were cloned into pGreenII0800-LUC. (**e**) Expression level of the selected 19 kDa zein genes in panels (**b**,**d**). ** indicates significance (*p* < 0.01) using the Student’s *t*-test.

The full-length *ZmThx20* ORF without the translation stop codon was cloned and then introduced into the pUC18-P35S::*GFP*-Tnos vector. The recombinant plasmid was introduced into maize leaf protoplasts using the PEG–calcium. A transient assay in maize protoplasts indicated that ZmThx20 is a nuclear-localized protein (Figure 7a).

To explore the downstream regulatory genes of *ZmThx20*, we used PlantPAN3.0 (http://plantpan.itps.ncku.edu.tw (accessed on 8 November 2021)) to predict the binding sites to analyze the downregulated genes in the transcriptome. Next, we analyzed the 0–600 bp promoter sequences upstream of several downregulated (*sh2008*/WT) genes. To this end, we selectively cloned several 19 kDa and 22 kDa zein promoter sequences and connected them into the pHis2 vector, and the CDS region of *ZmThx20* was inserted into the pGADT7-rec vector for yeast strain YM4271 transformation. The results indicated that the transcription factor could bind to the promoter sequence (Figure 7b). Dual-luciferase (LUC) assays also showed that ZmThx20 improved the transcriptional activity of these genes (Figure 7c). It was concluded that the ZmThx20 could regulate the expression of some zein genes and affect the synthesis of proteins in grains.

## 3. Discussion

In previous reports, the shrunken mutation was mainly caused by a deficiency in the enzymes related to abnormal starch syntheses, such as *sh2* and *bt2*, which abolished the conversion of UDP-glucose to ATP-glucose [54,55,56] and *su1*-blocked starch debranching. Abnormal levels of zein protein synthesis, cleavage, localization, and storage will lead to significant morphology and content changes in the kernels, often an opaque or semi-opaque phenotype, and sometimes lead to the UPR [39,46]. In this study, an abnormal kernel development mutant with a shrunken and opaque kernel was identified based on natural variation. This kernel phenotype is due to the two major component changes in the endosperm, both the reduction of starch and storage proteins. The starch content in mature kernels of the *sh2008* mutant was 26% of that observed in the WT. In contrast, the soluble sugar content was significantly higher in the *sh2008* mutant during the kernel milk and kernel dough stages, which are the two important stages for starch synthesis and accumulation in the maize endosperm. The starch grains in *sh2008* were loose and had a less proteinaceous matrix surrounding them. When dry and matured, the infilling in the endosperm, mainly the accumulated starch, was not completely filled in the pericarp of *sh2008*. The total storage protein and major storage protein zeins in the kernels of *sh2008* were ~70% of that in the WT control, especially the 19 kDa α-zein and 22 kDa α-zein proteins. The contents of various amino acids changed significantly, with elevated aspartic acid, serine, alanine, beta-alanine, and GABA. Lysine is lower in the mutant, although this difference does not seem to be significant. In contrast to the high lysine content in the *o2* mutant, the decreased lysine content was similar to that in the *fl4* semi-dominant opaque mutant [11], although not significant. In the WT, the protein bodies were oval and distributed uniformly around the starch granules, whereas in *sh2008*, the protein bodies were small and unevenly distributed. The reduction of the two major components in the endosperm led to shrunken and opaque kernels in the *sh2008* mutant.

Previous studies have shown that some trihelix transcription factors are involved in plant development and stress responses. A decrease in AtGTL1(AT1G33240) gene expression under drought stress conditions resulted in a reduction in stomatal number, which plays a role in water conservation [57]. AtGTL2 (AT5G28300) and AtGTL1 are genes related to the development of the epidermis, which can regulate the expression of cell cycle-related genes and result in a decrease in stomatal density under drought stress conditions [58]. The EDA31 gene (AT3G10000) has only one trihelix domain associated with embryo sac development during seed formation in Arabidopsis [59]. A gene of the SIP1 family (AT1G54060) was reported to be involved in the key regulation of early embryonic development [60] and seed saturation [61]. The rice SHATTERING1 (SHA1) gene is a member of the SH4 subfamily of the trihelix family and plays an important role in cell differentiation and activation. The *SHA1* mutant causes the disappearance of seed drops in cultivated rice [62]. Although the roles of trihelix family transcription factors have been discovered in light-relevant and other stress tolerance processes [63,64], their functions in maize kernel development have yet to be reported. In the trihelix transcription factors, *ZmThx20* was found to be involved in kernel development, although the mechanism remains unclear. *ZmThx20* belongs to a *Poaceae*-specific sub-group trihelix transcription factor and was predicted to be a member of the GT-2 trihelix family transcription factor based on the features of the trihelical DNA-binding domain. This sub-group trihelix transcription factors shared high similarity with the AtGT-2 sub-group (AtGT-2, AtDF1, and PTL) and AtGT-1 sub-group (AtGTL1, AtGT2L, and AT5G47600). Although the GT-2 members in Arabidopsis and rice were identified early by sequence homology, their function is still not clear except that AtDF1 is required for the synthesis of seed mucilage polysaccharides, PTL represses growth in Arabidopsis thaliana, AtGTL2, and AtGTL1 related to the epidermis development and EDA31 associated with embryo sac development. However, expression profiling analysis showed that GT-2 members might also participate in abiotic stresses. In this paper, the loss function of ZmThx20 led to elevated levels of alanine, beta-alanine, and GABA. The elevated beta-alanine and GABA serve as osmoprotective compounds and intercellular signaling molecules (GABA) [65,66]. However, the regulation mechanism is still not clear. The comparison of the DEGs, main enriched GO terms, and the targets from *sh2008* mutant versus WT and the result from O2 versus WT indicates that *ZmThx20* may function through, but not limited to, *O2* to control the expression of zein protein genes.

Comparative transcriptome analysis using the 15 DAP kernels from the *sh2008* and WT indicated that *ZmThx20* (or its transcriptional regulation complex) plays an extensive role in plant development and response to environmental stress. The top highly enriched GO terms were the responses to the stimulus, development, and metabolic processes. For zein protein content changes, the significantly downregulated zein protein-coding genes, especially the 22 kDa and 19 kDa zein genes, are probably the major reason. The fifty-seven DEGs involved in molecular function GO terms nutrient reservoir activity (GO: 0045735) were significantly reduced in *sh2008* compared to WT. For starch synthesis, the increased breakdown of sucrose and reduced starch synthesis genes may contribute to the reduction of the final starch accumulation in the *sh2008* mutant. *ZmThx20*-dependent transcriptional regulation is involved in maize kernel development.

## 4. Materials and Methods

### 4.1. Plant Materials

The maize (*Zea mays*) mutant *sh2008* was obtained from the natural variation of the maize inbred line DH4866 (Shandong Denghai Seeds Co., Ltd., Yantai, China). We used the *sh2008*/+ genotypes as males crossed into the B73, Q319, and W22 inbred lines to produce the F2 populations. The segregating F2 (B73 background) seeds were used for map-based cloning and histological observation at various days after pollination (DAP). Maize plants were cultivated in the field at the campus of Shandong University, Jinan, China.

### 4.2. Determination of Starch, Soluble Sugar, Amino Acid, and Protein Content in Maize Kernels (Endosperm)

The starch content in mature seeds was determined as previously described [67]. The following formula was used to calculate starch content: starch content (%) = G × 0.9/DW × 100%, where G denotes the total glucose content of the seeds and DW is the dry weight of the seeds. 

For the soluble sugar content measurements, the kernels were harvested from the segregating ears at 15, 20, 24, 27, and 34 DAP and then dried to a constant weight at 80 °C in a dryer. Soluble sugar content was measured as previously described [68].

Ten mature kernels of the WT and mutant were collected from segregating the F2 ear for each sample. The total proteins, zein, and non-zein proteins were extracted from 70 mg of dried endosperm flour, as previously described [31]. Seventy mg of powder was added to 1.0 mL of extracting solution (0.0125 M sodium borate (pH 10.0), 1% SDS, and 2% β-mercaptoethanol). After incubating in a shaker at 37 °C for 12 h, the sample was centrifuged for 15 min at 12,000 rpm in an Eppendorf microcentrifuge, and the supernatant was the total protein. We connected 300 μL supernatant with 700 μL absolute ethanol, mixed well at room temperature for 2 h, and then centrifuged again. The supernatant was zeins, and the pellet consisted of non-zein proteins. The supernatant and precipitation were freeze-dried in a freeze dryer, 200 μL IPG solution was added to dissolve, and it was stored in the −80 °C refrigerator. Protein quantification analyses of the total protein, zein, and non-zein fractions were performed using a BCA standard kit (DQ111-01; Transgene, Beijing, China). SDS–PAGE was performed on 12.5% polyacrylamide gels, and the gels were stained with Coomassie Brilliant Blue R250. All measurements were performed at least three times.

### 4.3. Light Microscopy, Scanning Electron Microscopy (SEM), and Transmission Electron Microscopy (TEM) Analysis of the Morphology and Structure of the Starch Granules and Protein Bodies

The WT and mutant kernels were harvested from the ears at 15 and 20 DAP, respectively. The cells were then fixed in 50% FAA (containing 50% ethanol and 4% formaldehyde) for 24 h. The samples were dehydrated in an ethanol gradient series (50, 70, 85, 95 and 100% ethanol), cleared with xylene, and embedded in paraffin. The samples were sliced to a thickness of 7 μm. The sections were stained with Schiff reagent and observed under a microscope (BX51; Olympus, Tokyo, Japan).

The starch granules of mature seeds of the WT and mutant were observed using scanning electron microscopy (SEM). The protocol was completed as previously described [69]. The top of WT and *sh2008* mature kernels were cut to break naturally, and the samples were dried in a drier and sprayed with gold by vacuum evaporators 108 (Cressington, Watford, England (UK)). The samples were then observed using a scanning electron microscope (FEG 250; Quanta, FEI, Columbia, MD, USA).

Immature WT and mutant kernels at 20 DAP were collected from the same segregating ear and cut to approximately one cubic millimeter in size at the endosperm margin and placed in 2.5% glutaraldehyde solution. The samples were stained with osmic acid and observed using transmission electron microscopy (Tecnai G2 F20, FEI, Columbia, MD, USA).

### 4.4. Subcellular Localization, Overexpression, and Gene Editing of ZmThx20

The full-length *ZmThx20* ORF without the stop codon was cloned and introduced into the pUC18-P35S::*GFP*-Tnos vector. The recombinant plasmid was introduced into maize leaf protoplasts with PEG–calcium [70]. A confocal laser scanning microscope (FV3000; Olympus, Tokyo, Japan) was used to detect the fluorescence signals of the protoplasts.

The ORF of *ZmThx20* was cloned and integrated into the modified plasmid vector pCAMBIA3300 (P35S::*ZmThx20*-Tnos-P35S::*bar*-Tnos) using the *Sam*I and *Kpn*I restriction sites. An sgRNA that directly targets sequences located at nucleotides 1495–1517 of *ZmThx20* was produced and cloned into the pVK005-2 vector (Beijing v-solid Biotechnology Co., Ltd. Beijing, China) using the *Asc* I and *Spe* I restriction sites. The *sh2008* mutant in immature embryos and maize inbred line Q319 embryos were used as the genetic transformation receptors. Resistant calli were screened on a glyphosate-selective medium (10 mg/L). After the PCR assay for transgenes, the transgenic plants were transplanted into a greenhouse for propagation.

### 4.5. qRT-PCR and Transcriptome Analysis

Total RNA was extracted from the plants and kernels of the WT and *sh2008* mutants using TRIZOL reagent (Sangon Biotech, Shanghai, China). For qRT-PCR, about 500 ng of total RNA was used for first-strand cDNA synthesis using the RT reagent Kit (Takara, Kyoto, Japan). All qRT-PCR analyses were carried out in a LightCycler^®^ 96 system with SYBR^®^ Green Premix Pro Taq HS qPCR Kit (AG11701, Accurate Biology, Changsha, China). The maize *ZmTubi* gene was used as an internal control. The gene expression levels were analyzed using the 2^−^^∆∆^^Ct^ analysis method. All primer sequences used for qRT-PCR are listed in Table S1.

Three biological replicates of the RNA samples were collected from WT and *sh2008* mutant kernels (DAP15), and six libraries were constructed and sequenced using the BGISEQ-500 platform (BGI, Wuhan, China) (http://www.seq500.com, (accessed on 8 November 2021)). For gene expression analysis, the matched reads were calculated and then normalized to reads per kilobase of transcript per million mapped reads (RPKM) using RESM software. The significance of differential gene expression was confirmed with the BGI bioinformatics service using the combination of the absolute value of log_2_-ratio ≥ 1 and Q ≤ 0.05.

### 4.6. Yeast Single Hybrid Experiment

The ORF of *ZmThx20* was cloned and integrated into the plasmid vector pGADT7-rec (the primers used are indicated in Table S1). The promoter of 19 kDa zein was cloned and integrated into the plasmid vector pHis2 (the primers used are indicated in Table S1). We transformed the plasmid pairs into yeast strain YM4271 and placed them on the SD/-Leu/-Trp plate. The positive monoclonal colonies were screened, diluted in equal proportions after shaking, and then dropped on SD/-Leu/-Trp/-His+3AT (150 mM) medium. Growth status was observed and photographed after 2–3 days.

### 4.7. Transient Transcription Dual-LUC Assay

Dual-LUC assays using *N. benthamiana* plants were performed as previously described [71]. Briefly, the full-length ORF of *ZmThx20* was fused into the overexpression vector pCAMBIA3300 (P35S::*ZmThx20*-Tnos-P35S::*bar*-Tnos). For the reporters, the promoters of 19 kDa zein were cloned into pGreenII0800-LUC (the primers used are indicated in Table S1). A TransDetect Double-Luciferase Reporter Assay Kit (TransGen Biotech) was used to measure Renilla and firefly activity according to the manufacturer’s instructions.

## 5. Conclusions

In this study, we characterized a maize shrunken kernel mutant, *sh2008*, which exhibits a delayed embryo and endosperm development phenotype. To understand the molecular basis of this mutant, we isolated the *sh2008* gene by map-based cloning and found that it encoded a member of the trihelix family transcription factor *ZmThx20*. Through the analysis of biochemical components, we found that the *ZmThx20* mutation caused a sharp decrease in the synthesis and accumulation of major nutrients in grains and a porous structure on the surface of starch grains. Transcriptome analysis of the kernels from *sh2008* and WT showed that the GO terms of translation, nutrient reservoir activity, etc., were enriched in the down-regulated DEGs of *sh2008*/WT. Although some transcription factors have been reported in maize, our study provides a new transcription factor that plays an essential role in maize seed development.

## Data Availability

RNA-seq data are available from the China National Center for Bioinformation (https://ngdc.cncb.ac.cn/ (accessed on 8 November 2021)), under the GSA accession number: CRA005024.

## Supplementary Materials

The following are available online at https://www.mdpi.com/article/10.3390/ijms222212137/s1.

## Abbreviations

kDa	Kilodalton
O2	Opaque 2
FL	Floury
OHP	O2-heterodimerizing proteins
PBF	DOF family TF Prolamin-box binding factor
*sh*	Shrunken
*su 1*	Sugary 1
WT	Wild-type
DAP	Days after pollination
GO	Gene ontology
DEG	Differentially expressed gene
qRT-PCR	Quantitative real-time reverse transcription-polymerase chain reaction
SEM	Scanning electron microscopy
TEM	Transmission electron microscopy
