# Supplementary material for: Trihelix Transcription Factor ZmThx20 Is Required for Kernel Development in Maize
Source: Int J Mol Sci. 2021 Nov 9;22(22):12137. doi: 10.3390/ijms222212137 (PMC8624104; doi:10.3390/ijms222212137)
Supplement: Supplementary file 1 [file ijms-22-12137-s001.zip › ijms-1429690-supplementary-for XML conversion.pdf]

International Journal of
Molecular Sciences

MDPI

Supplementary Materials

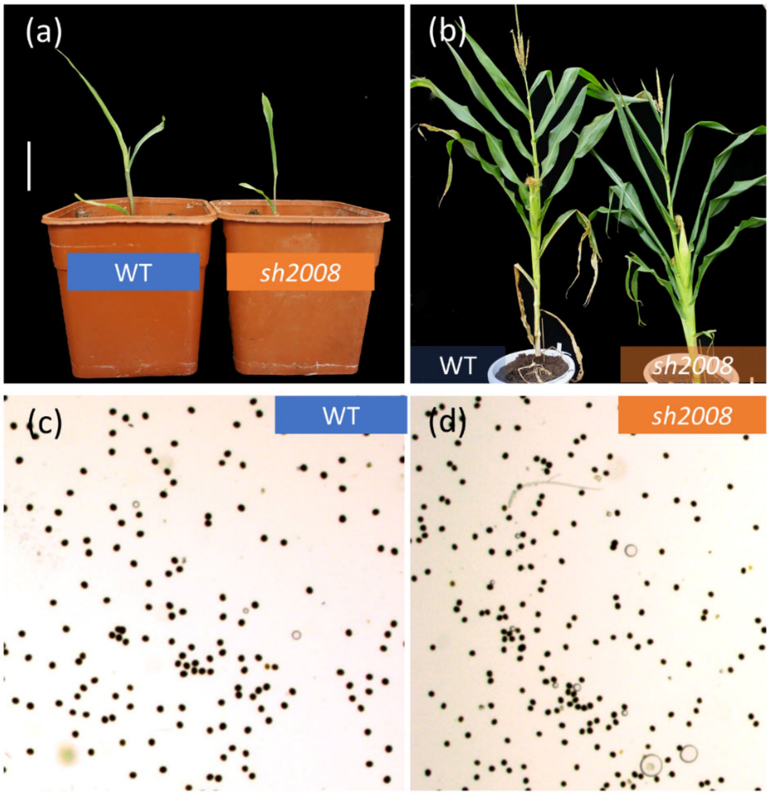

Figure S1. Morphology of seedlings, mature plants, and pollen viability from WT and *sh2008*. (**a**) Wild-type (WT, left) and mutant (*sh2008*, right) plants were transplanted into soil after rooting. Scale bar, 10 cm. (**b**) WT (left) and *sh2008* mutant (right) plants were transplanted into soil under normal management, both could produce tassel and ear under natural conditions. (**c**,**d**) The pollens of the WT and *sh2008* were stained I2-KI. Blue indicates the viability of the pollens; no significant difference was observed between them.

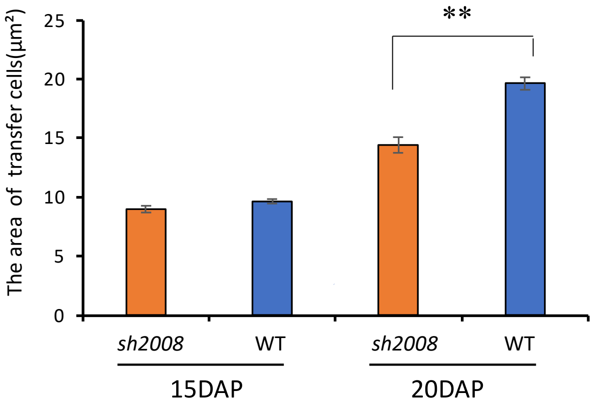

Figure S2. The area of basal endosperm transfer cells. Kernels at 15 and 20 DAP were used for paraffin sections as that in Figure 2 and the area of transfer cells was measured by ImageJ. Values are the means of the three replicates ± SD. ** denotes statistical significance with P<0.01 by using a *t*-test when compared with the value in the WT.

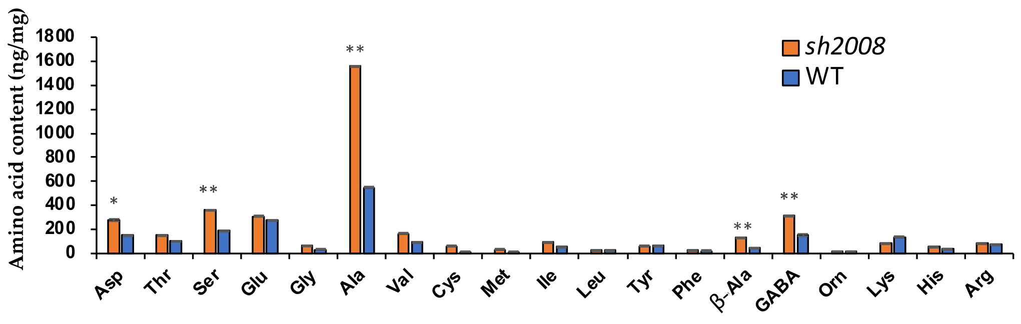

Figure S3. The amino acids content changes in the kernels of the WT and *sh2008*. The mature kernels were ground into a powder and then sieved with a 60-mesh sifter. Each sample was repeated in triplicate. *P<0.05, **P<0.01. Values are the means of the three replicates ± SD. ** denotes statistical significance with P<0.01 and *denotes statistical significance with P<0.05 by using a *t*-test when compared with the value in the WT.

	Chr	position_left	position_right	transcript_AGPv3	Whole_Seed					Whole_Seed							Embryo					Endosperm						
					2DAP	4DAP	6DAP	8DAP	10DAP	12DAP	14DAP	16DAP	18DAP	20DAP	22DAP	24DAP	16DAP	18DAP	20DAP	22DAP	24DAP	12DAP	14DAP	16DAP	18DAP	20DAP	22DAP	24DAP
	chr5	190228136	190232283	GRMZM2G169458_T01	302	364	306	323	319	142	136	81.3	78.1	68	47.6	31.6	1.4	0.08	0	0	0	11.8	7.24	0.45	19	0	0	0
	chr5	190228136	190232283	GRMZM2G169458_T02	12.4	26.2	8.52	18.4	8.74	1.32	16.2	9.73	8.39	3.65	3.38	4.46	0	0.55	0	0	0	0.29	2.92	6.81	8.34	0	0	0
	chr5	190236077	190237670	GRMZM2G169558_T01	22	11.4	16.2	14.3	17.6	13.3	7.9	4.92	27.2	16.3	9.56	12	7.99	16	0	0	0	5.65	0	7.17	9.31	0	0	0
1#	chr5	190240849	190244560	GRMZM2G169580_T01	25.7	31.6	19.9	27.9	29.7	27.7	45.2	79.1	65	74.1	68.7	88.7	89.1	70.6	69	62.5	57.5	35.8	70.9	107	76.2	95	84	112
	chr5	190475666	190477342	GRMZM2G117164_T01	14.3	27.5	35.6	34.1	31.4	17.4	23.2	4.06	12.5	10.5	4.72	5.08	0	0	0	0	0	0	0	0	0	0	0	0
2#	chr5	190481278	190485049	GRMZM2G117238_T01	0.4	0.02	0.04	0.02	0.01	0.06	0.85	0.12	0.05	0.05	0.05	0.61	1.51	0.04	0.82	0.03	0.01	1.96	6.3	0.3	0.77	0.09	0.29	0.25
	chr5	190481278	190485049	GRMZM2G117238_T02	34.3	21.3	16.5	21.8	26.7	46.9	49.9	27.6	26.4	17.4	15.4	18.3	65.1	67.2	34.9	23.5	17.8	81.1	46.2	35.5	30.6	20.9	16.2	18.9
	chr5	190554317	190561390	GRMZM2G072865_T01	10.8	23.3	11.6	12.3	20.6	11.1	8.13	4.79	4.08	4.98	5.44	12.6	5.05	8.65	11.7	6.79	8.33	16	3.97	2.59	7.06	7.77	3.84	5.7
3#	chr5	190554317	190558019	GRMZM2G072865_T02	1.47	9.44	0	3.35	5.64	2.59	2.63	4.29	3.06	3.9	2.29	5.77	2.55	0.66	3.52	3.06	2.91	6.67	0.83	1.1	1.98	5.67	2.11	3
	chr5	190554317	190558019	GRMZM2G072865_T03	44.1	5.94	47.4	20.2	3.27	22.4	24.6	17.2	31.6	19.6	7.99	21.7	14.8	0.01	18.7	11.6	42.5	33.2	18.4	7.37	13.8	29.4	36.2	23.7
	chr5	190554317	190558019	GRMZM2G072865_T04	35.9	45.7	0	41	39.3	41.2	38.7	34.1	34.2	51.3	72.8	43.5	50.2	53.1	38.9	46.3	29.3	10.3	26.6	30.5	41.1	33.1	42.6	59.4
	chr5	190554317	190557492	GRMZM2G072865_T05	0.08	0	0	0.01	2.04	0.16	0.01	0.44	0.15	0.04	0.04	0.11	0.09	0	0.76	0.22	0.1	0.59	0.05	0.02	0.4	0.22	0.02	0.04
	chr5	190554317	190555614	GRMZM2G072865_T06	0.33	0.13	0.21	0.09	0.77	0.11	0.17	0.55	0.1	0.07	0.05	0.08	0.06	0.11	0.46	0	0.16	0.07	0.12	0.01	0.44	0.95	0.01	0.15
4#	chr5	190563131	190564028	GRMZM2G135385_T01	0	0	3.46	14	15.9	0	0	7.52	19.4	4.29	16.2	9.32	0	0	0	0	0	0	0	0	0	0	0	0
	chr5	190604967	190607988	GRMZM2G135291_T01	0	1.72	8.74	2.23	7.21	1.52	4.55	14.8	22.8	41.7	35.7	38.1	0	0.68	0	0	5.77	2.91	0	36.9	30	50.5	64.8	57.3
	chr5	190674765	190677896	GRMZM2G156599_T01	0	0	1.74	5.3	10.2	16.7	16.9	16.5	28.2	20.3	19.1	12.5	0	0	0	0	0	0	0	0	0	0	0	0
	chr5	190684155	190686510	GRMZM2G156585_T01	1.72	0.68	0.44	0.34	3.65	5.39	4.64	2.89	3.14	0	1.57	2.21	5.31	2.65	2.23	1.63	1.88	15.6	7.36	4.25	3.28	0	0	0

Figure S4. Expression pattern analysis of the 10 genes between marker M190-2 and M190-6. Expression pattern analysis of the 10 protein coding genes between marker M190-2 and M190-6 . Four had higher expression levels during the kernel development stage: 1#(GRMZM2G169580 (Zm00001d017420)), 2#(GRMZM2G117238 (Zm00001d017423), 3#(GRMZM2G072865(Zm00001d017424)), and 4#(GRMZM2G135291(Zm00001d017427)), labeled by boxes. The expression level are based on Stelpflug et al., 2016 [49] and Hoopes et al., 2019 [50].

>ZmThx20
MLHHHGGAGSPYMAPTTADTGMDPFSPTPATRPAVSVTAIPPPPTMQLRPAGAGPSADFEELPA
GGAGAGAAASLQDDDMQMQADYGASAAGASGSGGNRWPREETLALIRIRTEMDADFRNAPLK
APLWEDVARKLAGLGYHRSAKKCKEKFENVHKYYKRTKDAHAGRQDGKSYRFFSQLEALHAAAP
QPQPPSGMTTVQAGPHHPMALAWTAGPTALGPAAGAGLPDLSFSSMSGSESEYDSDDDDAGEE
GLGRGEYHREMMAIFEGMMKQVTDKQDAMQRVFLETLERWEAERTAREEAWRRQEVARMNR
ERERLARERAAAASRDAALIAFLQCVGGGQGQPVRLPPHSAGASVVPAPPKPDCAPPSPRLDAAA
TSLQQLVPAQLKAVEALAWAGGEGGGSTSSRWPKEEVEALIQVRNEKDEQYHDAGGKGPLWEDI
AAGMRRIGYNRSAKRCKEKWENINKYYKKVKESNKRRPEDSKTCPYFHQLDAMYRKKHRGDRGRIT
AAGPNMQDSPSQRELEGKSSNDVDIDNRKSDEQGNVHTSPVSGNRDTAPTTTRPPGDGAKNKTAE
DNLKRTNVQLKQQQHEFGTDETDSDDDMARNHTAYTEEDDDEAKIKYKMDFQNPNMIGSSSNMS
APPPPAAAAATTAAAPTTSAAPTSNTFLAVQ

>zmthx20
MLHHHGGAGSPYMAPTTADTGMDPFSPTPATRPAVSVTAVPPPPTMQLQPAGAGPSADFEELPA
GDAGAGAVASLQDDDMQMQADYGASAAGASGSGGNRWPREETLALIRIRTEMDADFRNAPLK
APLWEDVARKLAGLGYHRSAKKCKEKFENVHKYYKRTKDAHAGRQDGKSYRFFSQLEALHAAAP
QPQPPSGMTTVQAGPHHPMAMAWTAGPTALGPAAGAGLPDHSFSSMSGSESEYDSDDDDAG
DEGLGRGEYHREMMAIFEGMMKQVAEKQDAMQRVFLETLERWEAERTAREEAWRRQEVARM
NRERERLARERAAAASRDAALIAFLQRVGGGQGQPVRLPPHSAGASVVPAPPKPDCAPPSPRLDA
AATSLQQLVPSPLKAVEALAWAGGEGGGSTSSRWPKEEVEALIQVRNEKDEQYHDAGGKGPLWE
DIAAGMRRIGYNRSASGARRSGRTSTSTTRRRRATRGAPRTPRPALTSTSSTPCTARNTVAAEAE-

Figure S5. Sequence and conserved domain analysis of *ZmThx20* in WT and *sh2008*. The wild-type and mutated *ZmThx20* were amplified and sequenced by using Sanger sequence. Translated amino acids are deduced from the sequence result. The conserved domain (two trihelix) was highlighted in yellow and green. The key "W" was highlighted in pink. The blue highlights the fourth amphipathic a-helix, with the general sequence (F/Y)- (F/Y)-X-X-(L/I/M)-X-X-(L/I/M). In the mutated *sh2008*, protein translation was terminated prematurely.

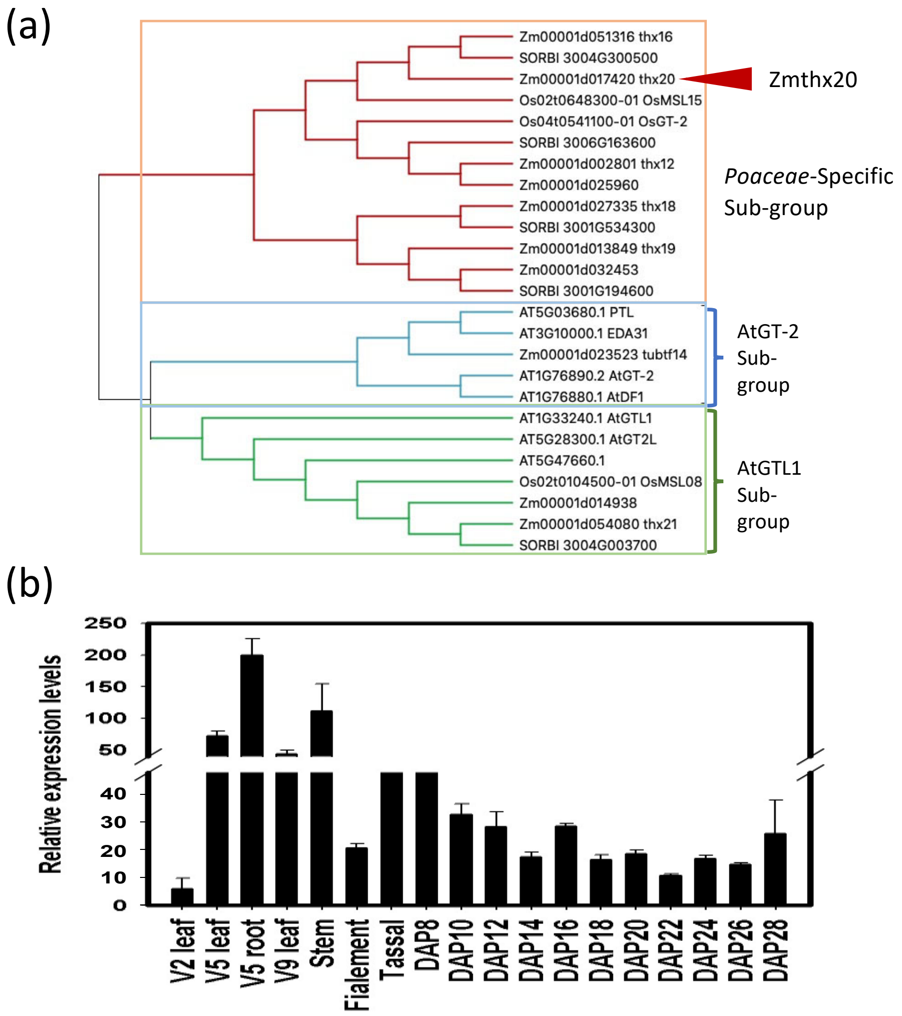

Figure S6. Sequence similarity and expression analysis of *ZmThx20.* (**a**) Sequence similarity analysis of *ZmThx20* and its homologs in other organisms. The evolutionary history was inferred by using the Maximum Likelihood method and JTT matrix-based model [S1, S2]. The phylogeny reconstruction was conducted in MEGAX. (**b**) qRT-PCR analysis of *ZmThx20* in various tissues and the developing kernels at different days after pollination (DAP). Maize *ZmTubi* was used as a reference gene. The relative gene expression levels were calculated using the $2^{-\Delta\Delta Ct}$ method[S3].

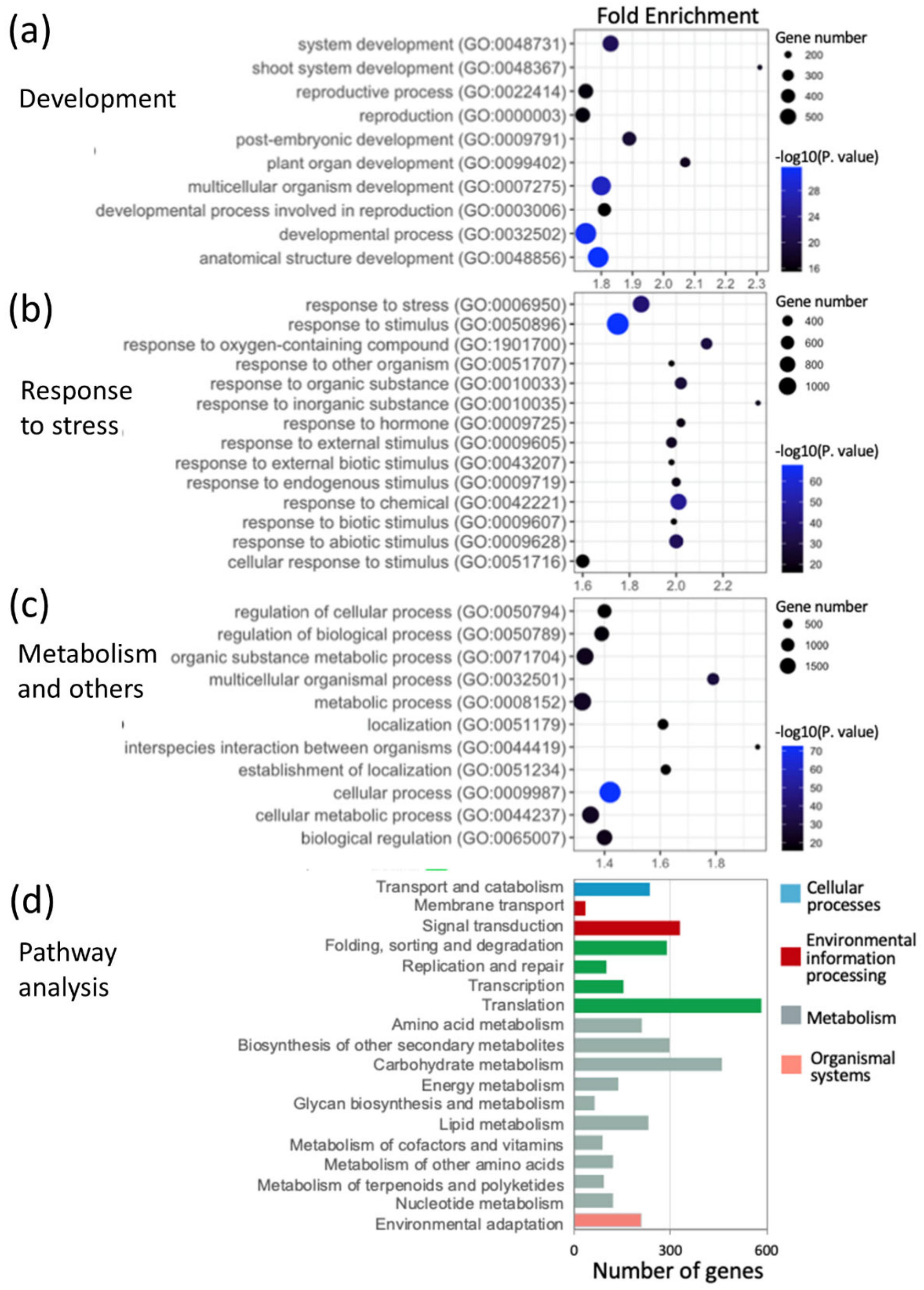

Figure S7. GO terms and pathway analysis of DEGs in kernels at the 15 DAP stage comparing *sh2008* mutant and WT. Bobble plot showing the fold enrichment, gene number, and p-value for each enriched GO term comparing *sh2008* and WT in development (**a**), response to stresses (**b**), metabolism, and others (**c**). (**d**) Pathway analysis of DEGs in kernels at the 15 DAP stage in the comparing *sh2008* mutant to WT. Gene numbers were mapped to KEGG (https://www.kegg.jp/kegg/pathway.html). The key DEGs used in (d-e) are listed in Supplementary Table 4.

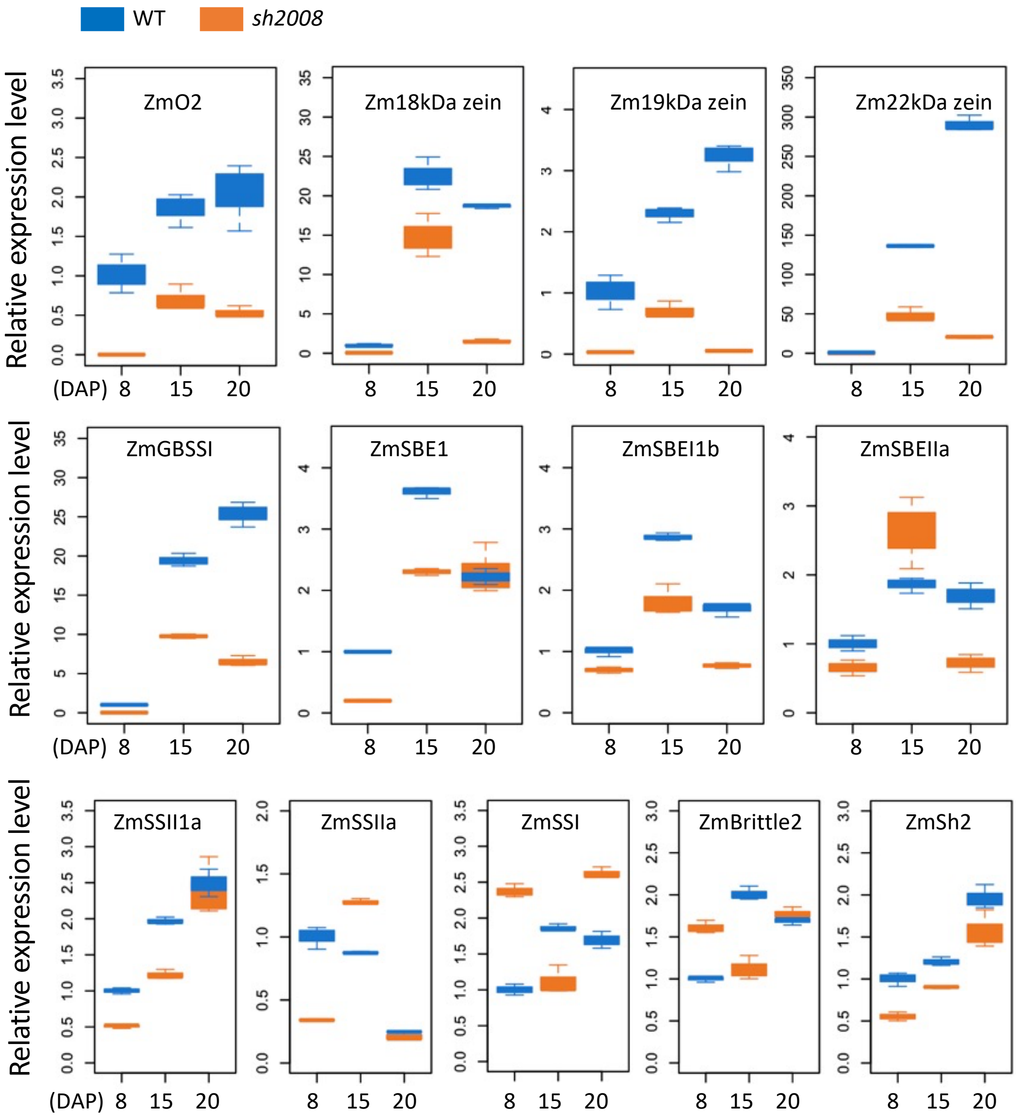

Figure S8. qRT-PCR analysis of *ZmThx20* and key DEGs in the kernels of WT and *sh2008* to validate the key DEGs identified by RNAseq.

Zmo2 (Opaque endosperm2, GRMZM2G015534/ Zm00001d018971), Zm 18kDa zein (delta zein structural 18, GRMZM2G100018/ Zm00001d037436), Zm19 kDa zein (GRMZM2G008913/ Zm00001eb171940(V5)), Zm22 kDa zein (GRMZM2G044625), ZmGBSSI (Granulae-bound starch synthase 1 (waxy1), GRMZM2G024993/ Zm00001d045462), ZmSBEI (starch branching enzyme IIb interacting protein1, GRMZM2G089553/Zm00001d011685), ZmSBEIIb (starch branching enzyme IIb interacting protein2, GRMZM2G091494/Zm00001d043703), ZmSEBIIa (starch branching enzyme IIb interacting protein1, GRMZM2G089553/ Zm00001d011685), ZmSSIIa (starch synthase 2 (sugary2), GRMZM2G348551/Zm00001d037234), ZmSSIIIa (starch synthase 3 (dull endosperm1), ZmSSI (starch synthase I, GRMZM2G129451/ Zm00001d045261), GRMZM2G141399/Zm00001d000002), ZmBrittle2 (brittle endosperm2,GRMZM2G068506/Zm00001d050032), ZmSh2 (shrunken2, GRMZM2G429899/Zm00001d044129). The maize kernels from the *sh2008* and WT plants were collected at DAP 8, 15, and 20, and RNA extraction and qRT-PCR were performed. Maize *ZmTubi* was used as a reference gene. The relative gene expression levels were calculated using the $2^{-\Delta Ct}$ method[S3], that is, $2^{-(Ct\ gene - Ct\ reference)}$, and the expression level at 8 DAP in WT was set as 1-fold.

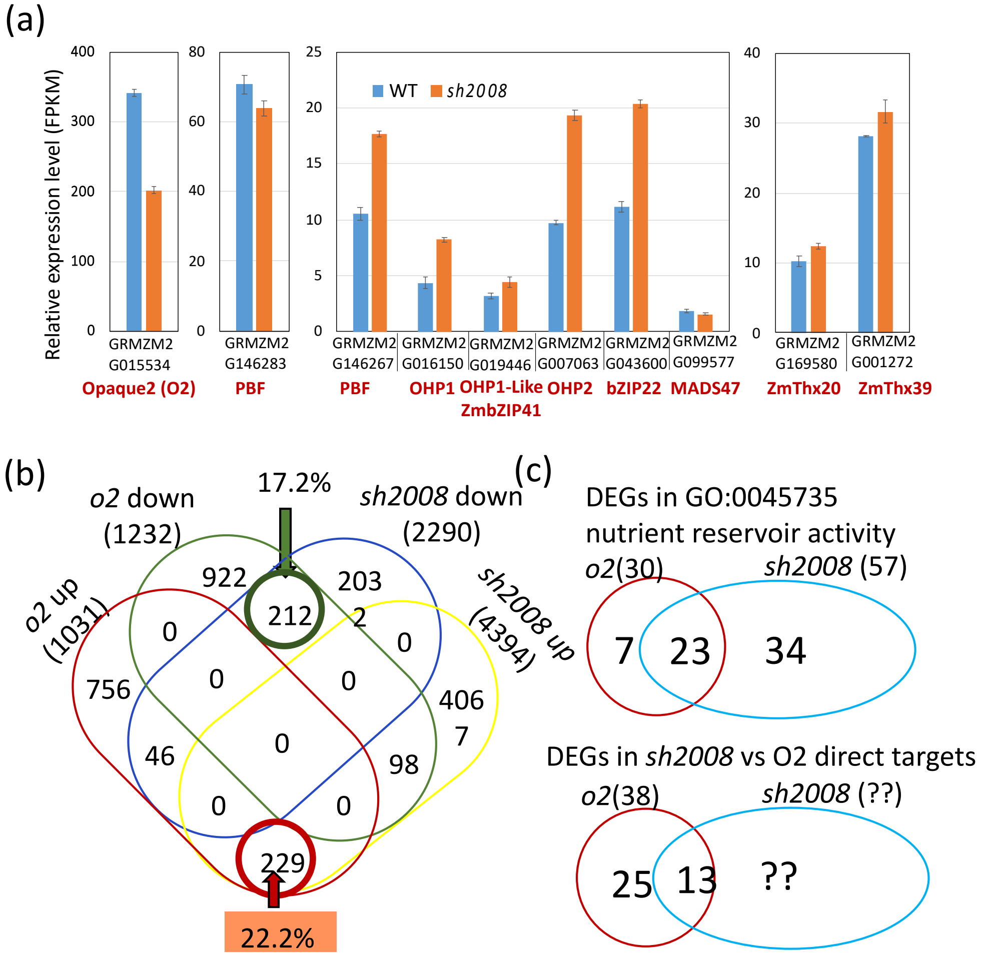

Figure S9. Comparison of *O2* and *ZmThx20* in gene expression. (**a**) Effect of *sh2008* on the expression of transcription factors involved in endosperm development. Bar plots show the relative expression levels (fragments per kilobase of transcript per million mapped reads) of Opaque2 (O2), O2-heterodimerizing proteins 1 (*OHP1*) and *OHP2*, DOF family TF Prolamin-box binding factor (*PBF*), *ZmbZIP22*, *ZmMADS47*, *ZmThx39*, and *ZmThx20*. (**b**) Comparison of *O2* and *ZmThx20* in gene expression for the overview DEGs. **(c)**, DEGs in nutrient reservoir activity (GO: 0045736) and O2 direct targets. The key overlapping DEGs in **(c)** are listed in Table S4.

References

S1. Jones, D. T.; Taylor, W. R.; Thornton, J. M., The rapid generation of mutation data matrices from protein sequences. *Computer applications in the biosciences : CABIOS* **1992,** 8, (3), 275-82.

S2. Kumar, S.; Stecher, G.; Li, M.; Knyaz, C.; Tamura, K., MEGA X: Molecular Evolutionary Genetics Analysis across Computing Platforms. *Molecular biology and evolution* **2018,** 35, (6), 1547-1549.

S3. Livak, K.J.; Schmittgen, T.D. Analysis of relative gene expression data using real-time quantitative PCR and the 2-ΔΔCT method. Methods 2001, 25, 402–408.
